# Applicability of the linear–quadratic model to single and fractionated radiotherapy schedules: an experimental study

**DOI:** 10.1093/jrr/rrt138

**Published:** 2013-12-17

**Authors:** Akifumi Miyakawa, Yuta Shibamoto, Shinya Otsuka, Hiromitsu Iwata

**Affiliations:** 1 Department of Radiology, Nagoya City University Graduate School of Medical Sciences, 1 Kawasumi, Mizuho-cho, Mizuho-ku, Nagoya, Aichi 467-8601, Japan; 2 Department of Radiation Oncology, Nagoya Proton Therapy Center, Nagoya City West Medical Center, 1-1-1 Hirate-cho, Kita-ku, Nagoya, Aichi 462-8508, Japan

**Keywords:** linear–quadratic model, biologically effective dose, hypofractionation, stereotactic radiotherapy, LQ model

## Abstract

The aim of this study was to examine the applicability of the linear-quadratic (LQ) model to single and fractionated irradiation in EMT6 cells. First, the α/β ratio of the cells was determined from single-dose experiments, and a biologically effective dose (BED) for 20 Gy in 10 fractions (fr) was calculated. Fractional doses yielding the same BED were calculated for 1-, 2-, 3-, 4-, 5-, 7-, 15- and 20-fraction irradiation using LQ formalism, and then irradiation with these schedules was actually given. Cell survival was determined by a standard colony assay. Differences in cell survival between pairs of groups were compared by *t*-test. The α/β ratio of the cells was 3.18 Gy, and 20 Gy in 10 fr corresponded to a BED_3.18_ of 32.6 Gy. The effects of 7-, 15- and 20-fraction irradiation with a BED_3.18_ of 32.6 Gy were similar to those of the 10-fraction irradiation, while the effects of 1- to 5-fraction irradiation were lower. In this cell line, the LQ model was considered applicable to 7- to 20-fraction irradiation or doses per fraction of 2.57 Gy or smaller. The LQ model might be applicable in the dose range below the α/β ratio.

## INTRODUCTION

With the development of stereotactic irradiation, various fractionation regimens, including single-fraction and hypofractionated regimens, are now used in clinics. Therefore, comparing the biological effectiveness of various fractionation regimens is necessary to evaluate the outcome of treatment. While the linear–quadratic (LQ) model (*n*_*2*_*d*_*2*_/*n*_*1*_*d*_*1*_ = (1 + *d*_*1*_/[α/β])/(1 + *d*_*2*_/[α/β]), where *d*_*1*_ and *d*_*2*_ are fractional doses and *n*_*1*_ and *n*_*2*_ are fraction numbers) and the biologically effective dose (BED) concept derived from the LQ model (BED = *D*(1 + *d*/[α/β]), where *D* is the total dose and *d* is the fractional dose, are very useful for comparison among conventionally fractionated regimens [1–3], it has been pointed out that the LQ model does not fit well to single-fraction and hypofractionated regimens or to high-dose-per-fraction radiotherapy [1, 4–6]. More appropriate models for use in hypofractionated regimens are therefore currently being investigated by several groups [7–10]. At present, however, no new model has been proven to fit perfectly to high-dose-per-fraction irradiation.

While attempts should be made to establish better dose calculation models, a practical issue in clinics might be to determine the fractional dose levels to which the LQ model is applicable. Mathematical calculations and model estimations have been attempted to resolve this issue [11, 12] but, to our knowledge, no experiment has been conducted to address this issue directly. In this study, therefore, we carried out experiments to compare various fractionation schedules directly, and to evaluate the applicability of the LQ model and BED concept to 1- to 20-fraction irradiation.

## MATERIALS AND METHODS

### Cell line

The EMT6 mouse mammary sarcoma line was used. Characteristics of this cell line were described in detail previously [13]. The cells were cultured in Eagle's minimum essential medium containing 12.5% fetal bovine serum. The cells were always kept in an exponentially growing phase and were subcultured on the day before experiments.

### Experimental design and procedures

It was planned to compare the effects of 1-, 2-, 3-, 4-, 5-, 7-, 10-, 15- and 20-fraction irradiation. First, the α/β ratio of the cell line was determined from four sets of single-dose experiments. Using the ratio, a BED for 20 Gy in 10 fractions was calculated, and fractional doses yielding the same BED were calculated using the LQ formula. Appropriate numbers of exponentially growing EMT6 cells were plated on 6-cm culture dishes in triplicate. Then, all of the fractionated irradiation schedules were carried out, and their effects on cell surviving fractions were compared. All irradiations were carried out using an X-ray machine (210 kVp, 10 mA, 2-mm Al filter) at room temperature, as described in detail previously [14]. The dose was calibrated using a RAMTEC 1000 dosimeter (Toyo Medic, Tokyo, Japan). The dose rate was 2 Gy per min. Control (0 Gy) groups received sham irradiation.

Regarding the interfraction interval, it was assumed that 24 h for this cell line would correspond to 1 week in humans, because the cell cycle time of this cell line was 11 h *in vitro* [15]. The potential doubling time for the EMT6 tumor cells *in vivo* was 1.5 days [16], while the potential doubling time for most human tumors is in the range of 4–12 days [17–19]. Our previous experiment showed that sublethal damage repair (SLDR) was completed within 2 h in this cell line [14], so an interfraction interval of 3 h 25 min (= 24/7 h) was considered to be sufficient to allow full SLDR. Therefore, irradiation was started at 8:00 a.m. and repeated at an interval of 3 h 25 min. The fractionated irradiation stopped at 9:40 p.m. every day; the interval between 9:40 p.m. and 8:00 a.m. the next day was regarded as corresponding to the weekend break in humans.

### Cell survival assay

The effects of radiation were determined by the standard colony assay. Colonies were fixed and stained after 7 d for the control group. For irradiated groups, colonies were fixed after 7–10 d of culture depending on the radiation schedules, because fractionated radiation took 4 d at most. After irradiation, colony sizes were visually evaluated, and they were fixed when the mean colony sizes were approximately 1.5 mm, similar to those of the control group.

### Statistical analysis

The LQ model fitting was performed using statistical software Mathematica (Wolfram Research, Inc., Champaign, IL, USA) as described previously [10]. All experiments were repeated four times, and differences in cell surviving fractions between pairs of fractionation groups were compared by *t*-test.

## RESULTS

**Fig. 1.**
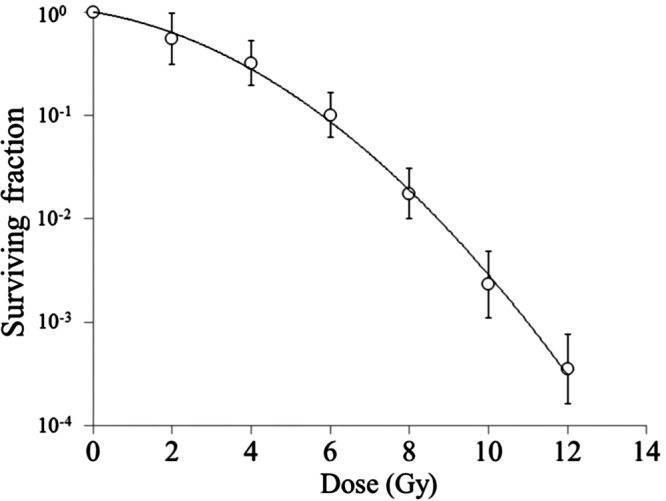
Dose–survival curve for EMT6 single cells. Bars represent standard deviation.

Figure 1 shows a dose-survival curve for EMT6 cells. The curve was fitted by the LQ model. The equation of the LQ model for the dose-survival curve was S = exp(−0.1411D − 0.0444D^2^). Therefore, the α/β ratio was 3.18 Gy (95% CI: 0.33–6.03 Gy). The BED for 20 Gy in 10 fractions was 32.6 Gy.

**Fig. 2.**
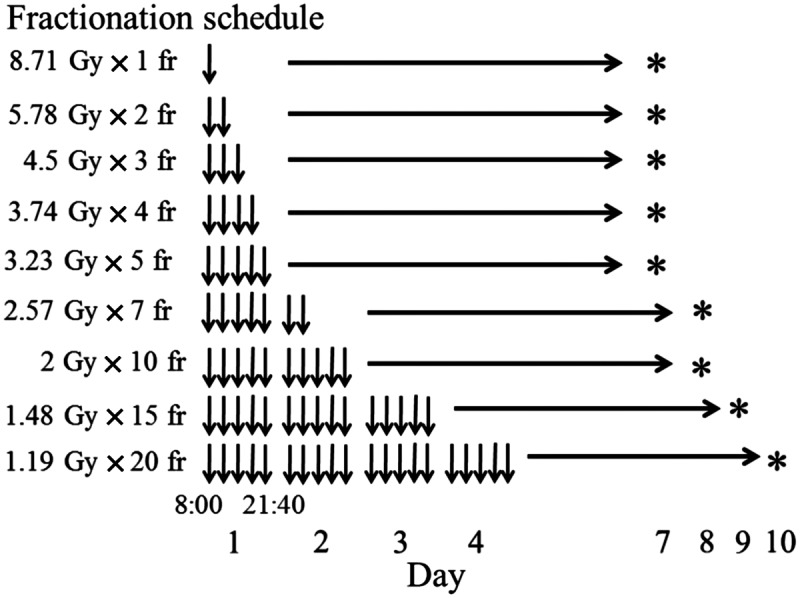
Radiation schedule. Irradiation was started at 8:00 a.m. and repeated at an interval of 3 h 25 min. Daily irradiation stopped at 9:40 p.m. *Colony staining.

**Fig. 3.**
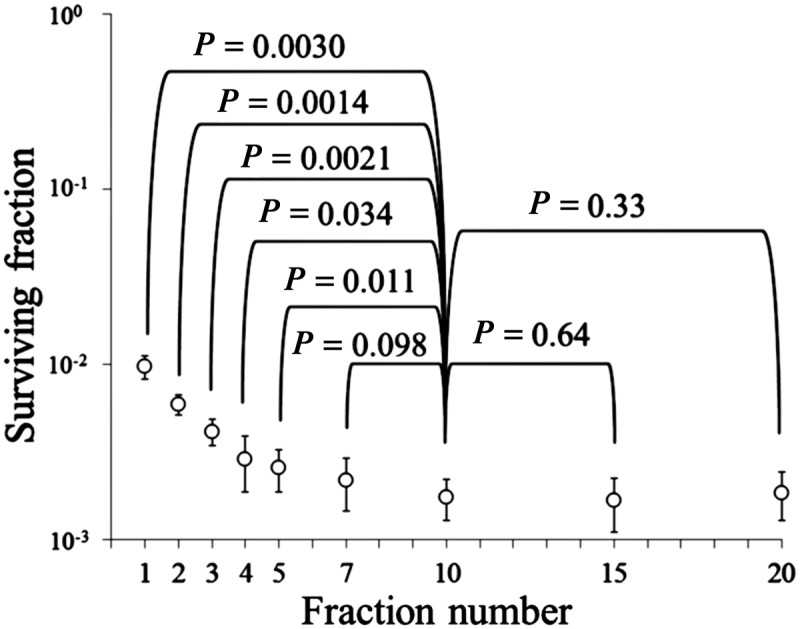
Surviving fractions of EMT6 single cells after single or fractionated irradiation with a biologically effective dose of 32.6 Gy for an α/β ratio of 3.18 Gy. Bars represent standard deviation.

On the basis of the α/β ratio of 3.18 Gy, 1- to 20-fraction irradiation to yield a BED_3.18_ of 32.6 Gy, as shown in Fig. 2, was performed. Figure 3 shows the cell surviving fractions after 1- to 20-fraction irradiation. Surviving fractions after 7-, 15- and 20-fraction irradiation did not differ significantly from the surviving fraction after 20 Gy in 10 fractions. On the other hand, surviving fractions after 1- to 5-fraction irradiation were higher than those after 10-fraction irradiation, and the discrepancy became greater with the decrease in the fraction number.

## DISCUSSION

In this study, the α/β ratio of EMT6 cells was experimentally determined to be 3.18 Gy, and 1- to 20-fraction irradiation to give a BED_3.18_ of 32.6 Gy was delivered to the cells. Despite the same BED, 1- to 5-fraction irradiation produced much higher cell surviving fractions than 10-fraction irradiation. Thus, it seems clear that the BED and LQ model are not applicable to single and hypofractionated irradiation in this cell line. On the other hand, they appear to be applicable to fractions of 7 or more.

To determine the applicable range of LQ formalism, it should be more pertinent to discuss the issue based on the dose per fraction rather than the fraction number. In this study, LQ formalism was applicable to 1.19–2.57 Gy per fraction. In a recent review, Shibamoto *et al*. [5] stated that the LQ model might be applicable up to a fractional dose approximately twofold the α/β ratio. The basis for this statement was as follows. Since the α/β ratio represents the dose at which cell killing from linear (α) and quadratic (β) components of the LQ formula is equal, the LQ model holds around the dose level of the α/β ratio. However, with an increase in the dose, the β cell kill component dominates in the LQ model, from which actual data have been shown to deviate. For this reason, it was considered that a fractional dose approximately twofold the α/β ratio might be a reasonable upper limit for the use of LQ formalism. However, our experimental results suggest that the LQ model is only applicable in the dose range below the α/β ratio. What are the reasons for the incompatibility of the model in the 1–5 fractions or at fractional doses of 3 Gy or higher?

When looking at the single-dose cell survival curve (Fig. 1), the LQ model appears to fit the data well. However, the surviving fraction at 2 Gy is slightly below the curve, while the surviving fractions at 4 and 6 Gy are slightly above the curve. This may partly explain the discrepancy between 2- to 5-fraction irradiation (i.e. 3.23–5.78 Gy/fraction) and 7 fractions or more (≤ 2.57 Gy/fraction). However, the dissociation of the single fraction (8.71 Gy) data cannot be explained. Another explanation may be the possibility of incomplete repair [1]. Wada *et al*. [20] recently reported that when the effects of potentially lethal damage repair (PLDR) and SLDR were taken into account in the LQ model, the cell survival response to carbon ion irradiation was well reproduced. However, PLDR may not be an important factor in the present study because we used exponentially growing cells. With respect to SLDR, we used an interfraction interval of 3 h 25 min. This was based on a previous experiment in which SLDR in this cell line was completed within 2 h [14]. On the other hand, completion of SLDR might take a longer time in other cell lines [21, 22]. Therefore, if it is assumed that the SLDR in our cell lines was not 100% complete within the interfraction interval of 3 h 25 min (although the magnitude was so small that this could not be proven experimentally), the effects of fractionated irradiation would become greater with an increase in the fraction number. However, the assumption of incomplete repair does not explain the similar cell survival fractions after 10-, 15- and 20-fraction irradiation in the present study. Another possible reason may be the cell cycle redistribution during fractionation. A longer cell cycle arrest is expected after a high dose than after a low dose, so a larger increase in radiosensitivity due to cell cycle redistribution may be expected when smaller doses per fraction are used [23]. There may be other problems intrinsic to the use of the LQ model in single and very hypofractionated irradiation, and this should be clarified in future studies.

If one dares to use the LQ model for single or hypofractionated irradiation, how large an error would be expected? The mean surviving fraction after a single 8.71-Gy dose in the fractionation experiment was 9.7 × 10^−3^ (log − 2.01). On the basis of the LQ equation, it is calculated to be 1.00 × 10^−2^, and the small difference would be within the range of experimental error. On the other hand, the mean surviving fractions after 7- to 20-fraction irradiation were 1.7–2.2 × 10^−3^ (log − 2.66 to − 2.78), and these surviving fractions correspond to those after a single dose of 10.3–10.5 Gy on the survival curve in Fig. 1. Therefore, the use of LQ formalism might lead to about 15–20% underestimation of the equivalent single dose. With an increase in fraction number, the discrepancy would decrease. Many clinicians still use the LQ model in hypofractionated radiotherapy without knowing the incorrectness of the model, and when one looks at such papers one should realize the magnitude of errors associated with the use of the LQ model from the above considerations.

## CONCLUSION

In conclusion, the LQ model is not applicable to single-fraction and hypofractionated irradiation. In the cell line investigated, the LQ model was considered applicable to 7- to 20-fraction irradiation or doses per fraction of 2.57 Gy or smaller. These results suggest that the LQ model conversion may be correct in the dose range below the α/β ratio. Further investigation of appropriate dose conversion models or methods in single and hypofractionated (five fractions or less) radiotherapy is encouraged.

## CONFLICT OF INTEREST

The authors declare that there are no conflicts of interest.

## FUNDING

This work was supported in part by a research grant from the Japanese Ministry of Education, Culture, Sports, Science and Technology.
